# Deep Breathing

**Published:** 1885-01

**Authors:** 


### DEEP BREATHING.

In this season, when coughs and colds are "all the rage," any method of preventing them, and checking the first symptoms without drugs, may be of inestimable value. Therefore the following suggestions are offered.

When you find you have a cough, and before it gets to be deep-seated, go into the air and practice deep breathing. Draw air into the lungs until they are completely distended, raising the arms above the head during inspiration to more fully expand the chest. Hold the air in the lungs for a few seconds, then breathe it out slowly. Repeat the operation a dozen times or more, and after an hour try it again.

Persistence in this treatment will often cure a newly-contracted cough in a few hours. If the cough is of long standing, pain may be felt under the shoulder-blades and across the chest during the breathing, but as this is caused by the tearing away of adhesions of the lung tissue, it will usually pass away in a day or two, and the fact that it is felt shows that the lungs need thorough inflation.

Three cases have recently come under our observation where this treatment has proved beneficial.

The first was that of a lady who had been troubled with a dry cough for several months, but whose lungs were apparently sound. In three days she cured herself entirely by deep breathing, and, although a month has gone by since then, there has been no return of the cough.

The second was a gentleman who thought his lungs were failing. Deep breathing gave severe pain, as above described, but it soon passed away. A burning sensation was also felt in the lungs at each deep breath, owing to the access of oxygen to irritated lung-tissue. The cough decreased in frequency and violence, he has gained in general health, and recovery will probably ensue.

The third was the Editor of the Journal of Health. He "caught cold," which settled into a severe cough. A dozen inhalations would stop the cough for an hour or two, when it would return and be stopped again in the same way. Two days' treatment drove it away entirely.

Sometimes the first deep breath is interrupted by a cough, but after a trial or two the inclination to cough can be controlled, and after five or six breaths are taken a sense of relief is felt and the desire to cough passes away.

A physician friend informs us that he has seen many cases of supposed consumption speedily cured in this way. At all events, it can do no harm to try it, and benefit may result.

